# Reliability of joint position sense measured in the knee using the level function of the iPhone “Measure” application

**DOI:** 10.1371/journal.pone.0256561

**Published:** 2021-08-27

**Authors:** Yuki Nakashima, Daisuke Iwaki, Toshihiro Kawae, Kenichi Fudeyasu, Hiroaki Kimura

**Affiliations:** 1 Division of Rehabilitation, Department of Clinical Practice and Support, Hiroshima University Hospital, Hiroshima, Japan; 2 Department of Physical Therapy, Makuhari Human Care Faculty, Tohto University, Chiba, Japan; 3 Department of Rehabilitation, Hiroshima University Hospital, Hiroshima, Japan; University of Innsbruck, AUSTRIA

## Abstract

An impaired joint position sense (JPS) causes activity limitations, postural imbalance, and falls. This study compares the reliability of knee JPS measurements between the iPhone’s “Measure” application and VICON motion capture system. Eleven healthy participants were recruited for the study. To conduct the study measures, the blindfolded participant, with an iPhone fixed to the lower non-dominant leg, was seated with their lower limbs in a relaxed position. The examiner held the participant’s leg at the target angle (30°/60° from initial position) for 5 s before releasing it. The participant was then instructed to move the leg to the same target angle and hold it for 5 s (replicated angle). Absolute angular error (AAE), i.e., the difference between the target and replicated angles, was measured. Intraclass and Pearson correlation coefficients established statistically significant relationships. The study comprised 6 males and 5 females of mean age 27.6±5.6 years, mean height 1.67±0.10 m, and mean body weight 60.7±10.3 kg. Strong correlations existed between iPhone and VICON 30° (ICC = 0.969, r = 0.960, P < 0.001) and 60° AAEs (ICC 0.969, r = 0.960, P < 0.001). Bland-Altman plots showed a mean difference of 0.43° and 0.20° between the AAE measurements at 30° and 60°, respectively. The iPhone’s “Measure” application is a simple and reliable method for measuring JPS in clinical practice and sports/fitness settings.

## Introduction

Proprioceptive sense helps an individual perceive the movement, action, and location of their body parts, and is composed of joint position sense (JPS), kinesthesia, and resistance sense [1]. Recent investigations have demonstrated that JPS is reduced in patients with anterior cruciate ligament (ACL) injury [2, 3], knee osteoarthritis [4], diabetes [5], and cerebrovascular disease [6]. Additionally, JPS is affected by aging and exercise habits even in the healthy population [7, 8]. The impaired JPS in patients with knee osteoarthritis and ACL injuries improves with exercise-based interventions [9–12]. Knee JPS has also been used as an outcome of exercise programs in the healthy population [13, 14]. Therefore, it is necessary to establish a simple and reliable method to measure knee JPS not only in patients with injuries but also in the healthy population.

JPS is usually monitored using the image-recorded angulation method or with an electrogoniometer, dynamometer motion chair, or 3D motion analysis system; however, these methods require special equipment and are costly [15–16]. Therefore, establishing a reliable and simple method to monitor JPS would determine the widespread application and effectiveness of new interventions for assessing JPS.

Recently, smartphones have become essential items in modern society and are used by many people; iPhones are of the most popular and widely used smartphones [17]. The use of smartphones in general clinical practice and sports/fitness settings in particular has increased [18, 19]. Previous studies have indicated that smartphones are excellent goniometric tools. Smartphone inclinometer applications and digital inclinometers are relatively inexpensive and easily available methods for measuring body movement [5, 20]. In addition, a method to measure knee JPS using an iPhone has been recently reported [21]; however, it requires a special application. The level function of the iPhone “Measure” application is a feature on all iPhones. As a free application and standard feature provided by Apple, it is compatible with the iPhone’s Operating System upgrade. Therefore, the iPhone’s “Measure” application was used in previous studies to measure the ankle joint angle [22, 23].

Cervical spine and hip angles measured using 3D motion capture, an expensive device with high reliability in measuring motion, and a smartphone inclinometer application were validated to be highly reliable [24, 25]. However, to the best of our knowledge, there are no reports comparing the reliability of knee JPS measured using a smartphone inclinometer with that measured using 3D motion capture. The purpose of this study was to determine the reliability of knee JPS measured using the level function of the “Measure” application on an iPhone. Our hypothesis states that measurements of JPS using the iPhone “Measure” application will correlate with those using the VICON motion capture system.

## Materials and methods

### Participants

All procedures were performed in accordance with the Declaration of Helsinki and were approved by the Hiroshima University Ethics Committee (approval number: E‐2219). All participants read and signed the appropriate informed consent form before the study commenced.

Eleven healthy participants were recruited from the medical staff of Hiroshima University Hospital for this study: 6 males and 5 females (mean age: 27.6 ± 5.6 years; mean height: 1.67 ± 0.10 m; mean body weight: 60.7 ± 10.3 kg). All participants were between the ages of 18 and 65 years; we conducted a survey of non-elderly participants because JPS has been reported to be decreased in the elderly [26]. Moreover, exercise habit has been reported to affect joint position perception [7, 8], but none of the participants had any exercise habits. The exclusion criteria were the presence of any neurological disease and a history of any orthopedic disease of the knee. The sample size was calculated using intraclass correlation coefficients (ICCs) to ensure that the number of participants in this reliability study was sufficient [27, 28]. The sample size was calculated to be 11 (minimum acceptable ICC, 0.50; expected ICC, 0.90; number of raters, 2; significance levels for a two-tailed test, α = 0.05 and β = 0.2) [29].

### Procedure

In this study, the JPS measurements were compared between two tools. Knee JPS measured using the level function of the iPhone (iPhone SE^®^, Apple Inc, CA, USA) “Measure” Application was tested against simultaneous measurements using the angle measurement function of VICON motion capture system (VICON, Oxford Metrics ltd., Oxford, UK). Although the two systems were used simultaneously to record JPS, they were not synchronized. All JPS measurements were performed at Hiroshima University Hospital. VICON is reported to be highly accurate, with a positional error of less than 2 mm and an angular error of less than 1.5° [30, 31]. The level function of the iPhone “Measure” application measures angles like an inclinometer.

**Fig 1:**
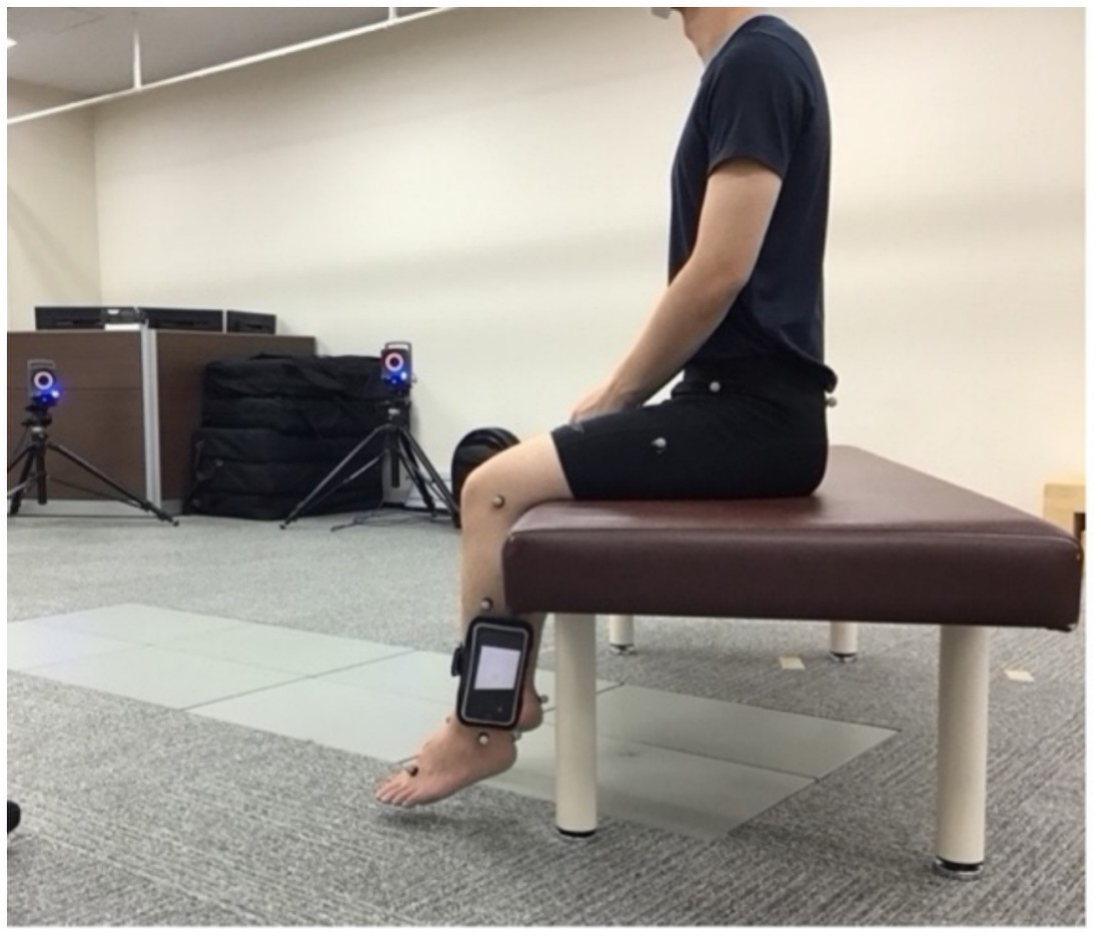
The knee JPS was measured on the non-dominant leg using VICON and an iPhone attached to the leg with a Velcro band. JPS: joint position sense.

Initially, the participants were seated with their lower limbs in a relaxed position (Fig 1) on a medical treatment table, from which their feet could not touch the ground [3]. The participants attached the iPhones to their non-dominant lower legs using a case with a Velcro band. The iPhone was positioned such that its bottom was at the level of the lateral malleolus. The longitudinal axis of the iPhone was aligned with the long axis of the fibula [5]. The participants’ legs were relaxed to set the iPhone measurement value to 0°, with their legs hanging down. All participants were accustomed to JPS testing procedures through explanations, demonstrations, and opportunities to practice. Practice trials were performed with dummy targets of any angle until all participants understood the protocol. JPS was measured using the passive-active JPS test in a sitting position.

The participants were blindfolded during the testing protocol. To maintain accuracy in measurements, all passive-active JPS tests were performed by the same examiner (YN). The data were obtained from VICON by DI and KF. Data were analyzed by YN. The leg used to kick the ball was defined as the dominant leg. Measurements were performed on the lower limb of the non-dominant leg. Because knee joint dominance affects knee JPS [32], we measured the non-dominant leg to standardize the measurement conditions. The test was performed under two conditions: 30° and 60° extension from the initial position. The order of the conditions was randomly selected. The examiner was instructed to position the participant’s leg at the target angle and hold it there for 5 seconds before returning to the starting position, and the participants were asked to concentrate and make note of their leg position. The participants were then required to actively move their leg to the target angle and hold it there for 5 seconds (replicated angle). Measurements were made thrice at each angle (30° and 60°) for each participant. The target positions were selected based on previously reported reliability and validity studies [8]. The protocol was the same for both target angles. All measurements in this study were performed in the evening.

The measurement screen of the iPhone was captured using an iPad fixed on a tripod. The video was copied to the PC and output at 30Hz was extracted using the video editing software, iMovie (Apple Inc, CA, USA); the joint angle was calculated by averaging the angles displayed on the iPhone screen for 3 seconds out of the 5 seconds that it was held stable. The angle from the iPhone was manually quantified by the examiner. The difference between the target and replicated angles reproduced by the subject (representing non-directional accuracy) was logged as the absolute angular error (AAE). AAE is often used as an indicator of JPS [8,15]. The test was repeated thrice and the average score was calculated. AAE was calculated using the following equation [33]:

$$\text{AAE}=\left(\left|\text{target}\;\text{angle}\;1-\text{replicated}\;\text{angle}\;1\right|+\left|\text{target}\;\text{angle}\;2-\text{replicated}\;\text{angle}\;2\right|+|\text{target}\;\text{angle}\;3-\text{replicated}\;\text{angle}\;3|\right)/3$$ (1)

A series of passive reflective markers were fixed on the principal anatomical landmarks of the lower limb and trunk according to the VICON Plug-In-Gait lower model (Plug-in-gait, VICON^®^, Oxford, UK). Passive reflective markers were attached bilaterally to the 1) anterior iliac spine, 2) posterior iliac spine, 3) lateral aspects of the thighs, 4) lateral femoral epicondyles, 5) lateral aspects of the shanks, 6) lateral malleolus, 7) calcaneus, and 8) second metatarsal head according to a commercially available kinematic model. The following anthropometric parameters were measured for each subject: height, body weight, the distance between the left and right anterior superior iliac spine (ASIS) points, leg length from ASIS to the medial malleolus, and the ankle and knee widths. The passive reflective markers were attached bilaterally. VICON data were sampled and analyzed at 100 Hz. The 2D flexion/extension angle of the knee joint was calculated from the lower leg and thigh segments.

### Statistical analysis

The numerical variables are presented as means and standard deviations (SDs), and the normality of their distribution was checked using the Shapiro–Wilk test. Two trial Bland-Altman plots were created for 30° and 60° AAEs to show the difference between the iPhone and VICON scores in each trial and the degree of agreement between the mean scores of the two trials.

The agreement between measurements was assessed by the ICC and its 95% confidence intervals (95% CI). This analysis was based on the two-way mixed effects, consistency, and multiple measurements model [34]. The relationship between the 30° and 60° AAEs calculated based on the iPhone and VICON measurements was examined using Pearson’s correlation coefficients. Standard error of the mean (SEM) was calculated using the following equation:

$$\text{SEM}=\text{sd}\sqrt{1}-\text{ICC}$$ (2)

The ICC values were interpreted as follows: >0.9, excellent; 0.9–0.75, good; 0.75–0.5, moderate; <0.5, poor [34]. The correlation values were interpreted as follows: >0.91, very strong; 0.9–0.68, strong; 0.67–0.36 moderate; 0.35–0.21 weak; <0.21 negligible [35]. P-values <0.05 were considered statistically significant. All statistical analyses were performed using IBM SPSS Statistics version 21.0 software (IBM, NY, USA).

## Results

**Fig 2:**
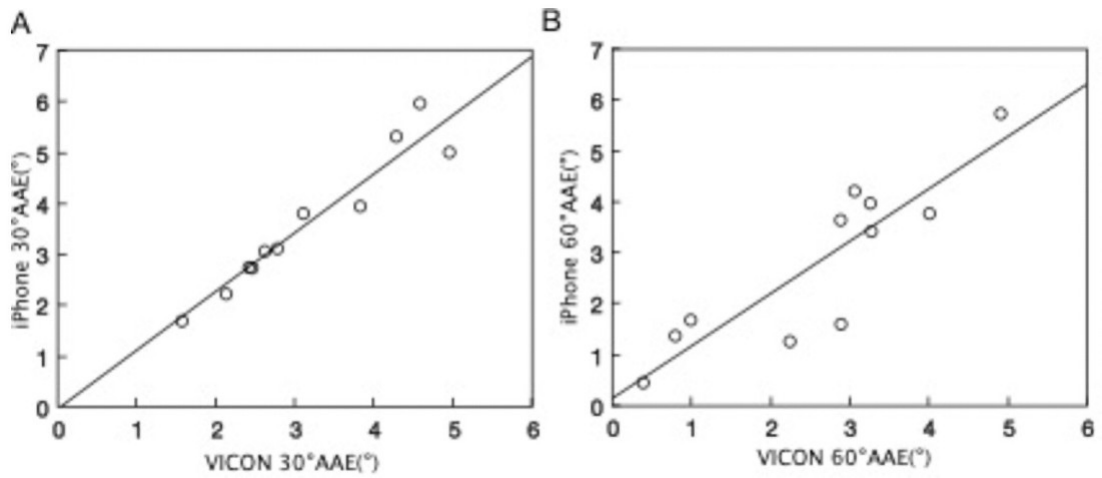
The relationship between AAE values of the iPhone and VICON. (A) 30°AAE (Pearson’s r = 0.960; P < 0.001). (B) 60°AAE (Pearson’s r = 0.879; P < 0.001). AAE: absolute angular error.

**Fig 3:**
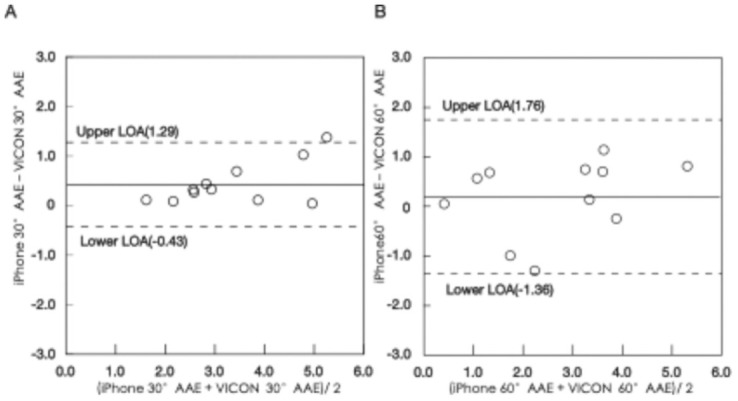
Bland-Altman plots. Mean values (continuous line) and LOA (dotted line) are shown on the Bland-Altman plots. AAE: absolute angular error; LOA: limits of agreement. (A) 30° AAE values measured with iPhone and VICON. Y-axis: Difference between the iPhone 30°AAE and VICON 30°AAE. X-axis: Average of both measures. Difference mean: Bias between the iPhone 30°AAE and VICON 30°AAE. LOA at 95% confidence intervals. (B) 60°AAE values measured with iPhone and VICON. Y-axis: Difference between the iPhone 60°AAE and VICON 60°AAE. X-axis: Average of both measures. Difference mean: Bias between the iPhone 60°AAE and VICON 60°AAE. LOA at 95% confidence intervals.

The means (SDs), ICCs, and correlation coefficients of the AAE measurements from the iPhone and VICON are shown in Table 1. The values were in excellent agreement, and there were strong correlations between both the iPhone and VICON 30° AAEs (ICC = 0.969, r = 0.960, P < 0.001) and 60° AAEs (ICC = 0.930, r = 0.879, P < 0.001). The relationship between the AAE values of the iPhone and VICON is shown in Fig 2. The average AAE values tended to be overestimated at both 30° and 60° for the iPhone compared with the VICON. The results of the Bland-Altman plot (Fig 3) show that the mean difference between two measurements at 30° AAE was 0.43° (limit of agreement, −0.43 to 1.29°) and at 60° AAE was 0.20° (limit of agreement, −1.36 to 1.76°).

**Table 1:** Concurrent validation of iPhone JPS measurements with VICON JPS measurements.

	iPhone	VICON	ICC (3, 2)	95% CI	SEM	r	P-value^†^
30°AAE	3.59 (1.35)	3.16 (1.10)	0.969	(0.883–0.992)	0.21	0.960	<0.001
60°AAE	2.82 (1.63)	2.62 (1.39)	0.930	(0.738–0.981)	0.39	0.879	<0.001

JPS: joint position sense; AAE: absolute angular error, which is the absolute value of the difference between the target and replicated angles, for both the iPhone and VICON, was calculated as the index of JPS; ICC: interclass correlation coefficient; CI: confidence interval; SEM: standard error of the mean. VICON motion capture systems are considered the gold standard. Values are presented as means (standard deviations).

^†^ Pearson correlation coefficient.

## Discussion

This is the first report to validate the reliability of knee JPS measured using the iPhone application against the gold standard for motion analysis, VICON. In this study, knee JPS values were collected simultaneously from the iPhone and VICON. Knee JPS measurements on the iPhone and VICON showed excellent agreement and strong correlation, suggesting that the iPhone measures knee JPS with an accuracy similar to that of VICON.

The results of this study showed that the inter-rater reliability (ICC = 0.930–0.969) of the iPhone and VICON was excellent, and measurement of knee JPS using the angle gauge, provided as a standard tool on the iPhone, was as accurate as that of the 3D motion analysis device. From our results, we observed that measurements at both 30° and 60° were accurate enough to be used in clinical measurements. It was confirmed that the AAE values, which act as indices of joint position perception, were consistent with that of previous studies. However, the results of ICC and correlation coefficients suggest that 30° AAEs may have a higher reliability than 60° AAEs, and that 30° AAEs have less bias in Bland-Altman plots. The ICC is excellent for both 30° and 60° AAE angles, but the error is considered to increase as the angle increases. Therefore, it may be optimal to measure at 30° AAE. The AAE values ranged from 2.82° to 3.59° in our study. This result was similar to the results of previous studies that measured AAE in a sitting position in healthy young participants (AAE: 2.2° to 4.9°) [8, 36–39], indicating that the iPhone-based method provides reasonable results. Moreover, in ACL-deficient patients, the mean difference in AAE values between the ACL-deficient and healthy participants groups was 5.3° [39]. Since clinically significant differences of at least 5° have been found in healthy participants [40], our method with a small standard error ranging from 0.21 to 0.39 may be useful. In our results, there was only a 0.2° to 0.43° difference between the mean values of AAE measured using the level function of the iPhone “Measure” application and VICON. Previously, the amount of change was reported to be 7.0°–11.3° after intervention using Kinesio taping, and we believe that the iPhone-based method can be used to evaluate these changes [41].

There have been several reports on using a smartphone for joint angle measurements, and good results in terms of reliability have been reported in a review article [42]. However, we were unable to obtain some of the applications used in the review. A method of measuring knee JPS with a smartphone fixed to the lower leg has been reported [5, 43]. Moreover, a previous study on measuring JPS with such a device was conducted using the shoulder joint to verify the reliability and reported a difference in JPS of 0.1°–0.4° with a magnetic tracking device [44]. However, knee JPS measurements using these methods have not been fully tested. This study validated a method of measuring knee JPS with an iPhone fixed to the lower leg, which showed good results.

There were some limitations to this study. First, only healthy participants were recruited. Since this study did not recruit from the clinical population, results cannot be extrapolated or used for ACL-deficient patients. Second, iPhone contact can decrease proprioceptive error because of additional information from the skin [33].

No previous studies have compared the accuracy of knee JPS measurements between smartphones and VICON. This study was, therefore, conducted in healthy young adults. However, the clinical use of the iPhone method requires validation in patients with prevalent diseases, such as ACL injuries and knee osteoarthritis. Accordingly, further studies will be planned in patients based on the results obtained in this study.

## Conclusion

The JPS measured using the level function of the iPhone “Measure” application correlated well with the values obtained using VICON system, the gold standard for JPS measurement via motion capture. This result suggests that the measurement of JPS using an iPhone has sufficient reliability for clinical practice. An iPhone can thus be used as a tool in general clinical practice and sports/fitness settings for measurement of JPS.

## Supporting information

S1 Data (XLSX)
